# The proportion of non-depressed subjects in a study sample strongly affects the results of psychometric analyses of depression symptoms

**DOI:** 10.1371/journal.pone.0235272

**Published:** 2020-07-06

**Authors:** Simon Foster, Meichun Mohler-Kuo

**Affiliations:** 1 Department of Child and Adolescent Psychiatry and Psychotherapy (KJPP), University Hospital of Psychiatry Zurich, University of Zurich, Zurich, Switzerland; 2 La Source, School of nursing sciences, HES-SO University of Applied Sciences and Arts of Western Switzerland, Lausanne, Switzerland; Mathematical Institute, HUNGARY

## Abstract

**Background:**

Recent studies have uncovered a peculiar finding: that the strength and dimensionality of depression symptoms’ inter-relationships vary systematically across study samples with different average levels of depression severity. Our aim was to examine whether this phenomenon is driven by the proportion of non-affected subjects in the sample.

**Methods:**

Cross-sectional data from the “Cohort Study on Substance Use Risk Factors” was analyzed. Self-reported depression symptoms were assessed via the Major Depressive Inventory. Symptom data were analyzed via polychoric correlations, principal component analysis, confirmatory factor analysis, Mokken scale analysis, and network analysis. Analyses were carried out across 22 subsamples containing increasingly higher proportions of non-depressed participants. Results were examined as a function of the proportion of non-depressed participants.

**Results:**

A strong influence of the proportion of non-depressed participants was uncovered: the higher the proportion, the stronger the symptom correlations, higher their tendency towards unidimensionality, better their scalability, and higher the network edge strengths. Comparing the depressed sample with the general population sample, the average symptom correlation increased from 0.29 to 0.51; variance explained by the first eigenvalue increased from 0.36 to 0.56; fit measures from confirmatory one-factor analysis increased from 0.81 to 0.97; the H coefficient of scalability increased from 0.26 to 0.48; and the median network edge increased from 0.00 to 0.07.

**Conclusions:**

Results of psychometric analyses vary substantially as a function of the proportion of non-depressed participants in the sample being studied. This provides a possible explanation for the lack of reproducibility of previous psychometric studies.

## Introduction

In recent years, a somewhat curious finding has emerged in studies on depression: both the strength and dimensionality of inter-relationships between depressive symptoms is influenced by the average level of depression severity observed in the study sample. For example, in two population samples of young adults from the USA and Switzerland: a) symptom correlations were strong in the general population, but surprisingly weak in those who were depressed; and b) the symptoms had a unidimensional factor structure in the general populations, but various multidimensional structures in those who were depressed [1]. In American and Dutch depression patients, Fried et al. found that while the average depression severity in the samples decreased over time, the average symptom correlation increased and the factor structure of the correlations became simpler [2]. Meanwhile, in American adolescent depression patients, Isa et al. found that while the average depression severity decreased over time, symptom correlations became stronger, the first eigenvalue and the total variance explained in the symptom correlations increased, the factor structure became simpler, and the measurement scale’s internal consistency grew stronger [3]. Other authors found that correlations between different depression scales increased substantially during clinical trials of depressed patients, while average depression severity on the same scales decreased [4, 5]. Finally, Beard et al. identified stronger overall connectivity of symptom networks when depression severity was decreasing in psychiatric patient samples [6].

Across a variety of samples and including both self-report and clinician-rated measurement instruments, the cited findings indicate that a study sample’s composition, in terms of its average level of depression severity, can have a substantial impact on the symptoms’ inter-relationships and on commonly-used psychometric models. Fried et al. explored a set of possible explanations for this finding, but concluded that no good explanation yet existed [2]. Our aim in the present study was to show empirically that the observed changes in depression symptoms’ correlations, dimensionality, and scalability can be explained at least partially by the proportion of non-affected participants in the sample. We evaluated two working hypotheses:

1. The magnitude of the symptom correlations varies systematically as a function of the proportion of non-depressed individuals in the sample. Specifically, a higher proportion of non-depressed participants leads to stronger symptom correlations.
2. Any change in the correlations’ magnitudes has a corresponding impact on all models that are rooted in the symptom inter-relationships. Specifically, the increases in symptom correlations result in a tendency towards unidimensionality, greater scalability, and greater network connectivity of the depression symptoms.

If these hypotheses hold, the implication is that dimensional, item response, and network models yield substantially different results, depending on sample composition. This would be a serious issue, since such analyses were based on rather different sample compositions [1–3, 6–16]. Hence, these studies might actually be incomparable. Furthermore, such a finding would raise the specter of having to select the ‘right sample’ for a given study purpose, implying that researchers would have to be mindful about sample composition when interpreting the results of previous literature or conducting their own research.

## Materials and methods

### Study design and participants

We analyzed cross-sectional data from the “Cohort Study on Substance Use Risk Factors” (C-SURF). C-SURF is a large cohort study of young Swiss men. Such an epidemiologic sample has the distinctive advantage of covering the full range of naturally-occurring variations in depression, thereby yielding results that should be generalizable to the general population of young men [17].

C-SURF was designed to be representative of non-institutionalized young Swiss men. Details on study enrollment, sampling and non-response bias have been published elsewhere by Studer et al. [18, 19]. The study protocol was approved by the Ethics Committee for Clinical Research at Lausanne University Medical School (protocol number 15/07). The study complies with the ethics standards of the national and institutional committees on human experimentation, and with the 2008 revision of the 1975 Helsinki Declaration. Informed written consent was obtained from all participants. Of the total of 5990 men who completed the baseline survey between September 2010 and March 2012, 132 (2.2%) were excluded due to missing values for depressive symptoms.

In order to assess the impact of the proportion of non-depressed individuals, we constructed a set of samples that contained an increasing proportion of non-depressed participants. First, we started with a study sample that only contained depressed participants. We then added a random sample of non-depressed participants that was 10% the number of depressed participants; then a random sample of non-depressed participants 20% the number of depressed participants; then 30%; and so on. In total, we added the following proportions of non-depressed participants to the original sample of depressed subjects: 0.10, 0.20, 0.30, 0.40, 0.50, 0.60, 0.70, 0.80, 0.90, 1.00, 2.00; 3.00; 4.00; 5.00; 6.00; 7.00; 8.00; 9.00; 10.00. Note that, in the 1.00-condition, we analyzed a sample that was composed of as many non-depressed as depressed participants; in the 2.00-condition, the sample contained twice as many non-depressed as depressed participants; in the 3.00-condition, three times as many; and so on. We completed the sample set by examining the original general population sample and the sample only containing the non-depressed participants.

### Measurement of depression

Self-reported depression symptoms were assessed via the Major Depressive Inventory–WHO-MDI [20, 21]. The WHO-MDI is a validated depression measure that asks about 12 symptoms over the past 14 days, using 6-point answer scales ranging from “never” (0) to “all the time” (5) for each symptom. The 6-point answer scales were used in all our analyses, rather than dichotomizing the symptoms into present or absent. Participants were classified as having no versus at least mild depression, as defined by Olsen et al., based on their MDI summation score [21]. Note that being classified as “no depression” did not necessarily imply being completely symptom free.

### Statistical analysis

Analyses were conducted using R version 3.3.2. Add-on packages that were used included “lavaan” for estimating polychoric correlations and confirmatory factor analysis [22]; “Mokken” for estimating Mokken scale analysis [23]; and “qgraph” for estimating symptom networks [24].

Correlation, dimensionality, scalability, and network analyses were carried out on the depression symptoms. The analyses were performed across sample sets with different proportions of non-depressed individuals (as described at the beginning of the Methods section), thereby showing how results changed as a function of the proportion of non-depressed participants. To ensure stable analyses, we repeated sampling from the non-depressed participants 500 times, and averaged analyses across these 500 samples. For example, the number of depressed participants was 356, meaning that 10% of this sample was 36. We therefore drew 500 samples of size 36 from the non-depressed participants, joined each one with the sample of depressed subjects, calculated symptom correlations in each of the 500 newly-generated samples, and then averaged each correlation across the 500 samples. The averaged correlations then provided our estimates of the symptom correlations when adding 10% non-depressed participants.

#### Symptom correlations

Since the depression symptoms had categorical scales, polychoric correlations were estimated [25]. Polychoric correlations estimate the correlation of two variables that are assumed to be continuous but that are measured on an ordinal categorical scale. Specifically, depression symptoms’ intensity is continuous but measured via a set of six ordinal categories (“never”, “some of the time”, up to “all the time”).

#### Dimensionality analysis

We used two commonly used approaches to assess the dimensionality of the symptom correlations, an exploratory and a confirmatory approach. In the exploratory approach, principal component analysis of the polychoric correlations was carried out, combined with parallel analysis (PA) [26]. Parallel analysis allowed us to determine how many principal components should be extracted. If symptoms are unidimensional, PA should suggest extracting one component.

Parallel analysis is a simulation-based method that compares the eigenvalues of the principal components derived from the actual sample against those obtained from a simulated data set of uncorrelated variables of the same size as the actual data set [26]. All principal components with eigenvalues larger than the eigenvalues found in the simulated data set were extracted. We generated 100 random data sets for each study sample by perturbing the original data [27], and then used the 95% percentile of this distribution as the threshold to be exceeded by the actual components.

We additionally calculated two indicators that should be higher if the symptoms tend towards unidimensionality: the amount of variance explained by the first eigenvalue, and the ratio of the first to the second eigenvalue. As a rule of thumb, if unidimensionality holds, the former should be higher than 20%, while the latter should be at least 3 [28].

The confirmatory approach consisted of performing confirmatory factor analysis (CFA), where all symptoms were modelled as indicators of one latent depression factor. The CFAs were estimated using mean and variance-adjusted weighted least squares estimates (WLSMV), which is the gold standard for categorical indicators [8, 25]. Model fit was quantified via standard criteria for good model fit [8, 9]: the Comparative Fit Index (CFI) ≥ 0.95; Tucker-Lewis Index (TLI) ≥ 0.95; and the Root Mean Square Error of Approximation (RMSEA) ≤ 0.05.

#### Item response analysis

We used Mokken scale analysis (MSA) for item response analysis [23]. MSA provides a set of criteria that allow for evaluating to what degree a set of items forms a hierarchical scale and was used to assess depression scales before [21]. MSA is non-parametric, meaning that the provided criteria are defined without assuming a particular form of relationship between the latent construct and the items (such as the logistic form assumed in parametric item response models).

We examined the scalability coefficient H, which indicates the overall quality of a scale [29]. The H coefficient can be calculated for individual items, as well as for the entire scale. The H coefficient of an item j can be interpreted as item j's discrimination index, whereas the H coefficient of the scale represents the average discriminatory power of the scale. As such, it is an index for the precision of ordering persons by means of their total scale scores [29].

#### Network analysis

Network analysis is a relatively new psychometric approach that conceptualizes depression as a causal system of interacting symptoms. The symptoms are represented as nodes of the network, whereas the relationships among the symptoms are represented as edges. Network analysis estimates the edges from the data, that is, the magnitude and sign (positive, negative) of the symptom relationships.

We estimated the network edges via Graphical Gaussian models, based on polychoric symptom correlations [6, 30]. These models can be interpreted as partial correlation models, wherein each network edge represents the relationship between the two symptoms when controlling for all other relationships in the network. Networks were regularized via the graphical lasso, resulting in sparse networks that explain the covariation among the symptoms with as few edges as necessary [6, 30].

## Results

Baseline characteristics of study participants are summarized in Table 1. Participants were young men (average age 20 ± 1.2 years) and roughly half of them had completed their secondary education. Almost 55% were French-speaking. The prevalence of depression of at least mild degree was 6.1%. Depression was slightly more prevalent in those who were older (6.7% versus 5.4%, p = 0.037) and among French-speaking participants (6.7% versus 5.3%, p = 0.025).

**Table 1:** Baseline characteristics of study participants.

	n (%)	Depression (prevalence in %)	χ^2^ (df) ^a^	p-value
Total	5858	6.1	-	-
Age (20 ± 1.2 years)				
Below median of participants	2951 (50.4)	5.4	4.3 (1)	0.037
Above median of participants	2901 (49.6)	6.7		
Education				
Primary school	2829 (48.4)	5.9	0.5 (3)	0.91
Secondary vocational education	1861 (31.9)	6.0		
Secondary school education	1051 (18.0)	6.6		
Above secondary	99 (1.7)	6.1		
Linguistic region				
German-speaking	2647 (45.2)	5.3	5.0 (1)	0.025
French-speaking	3211 (54.8)	6.7		

Note that per sociodemographic variable, additional participants may have been excluded from the analytic sample due to missing values in the sociodemographic variable.

^a^ Pearson’s Chi^2^-test for contingency tables with degrees of freedom (df). Tests compare the depression prevalence of subgroups of age, education, and linguistic region, respectively.

### Symptom correlations

**Fig 1:**
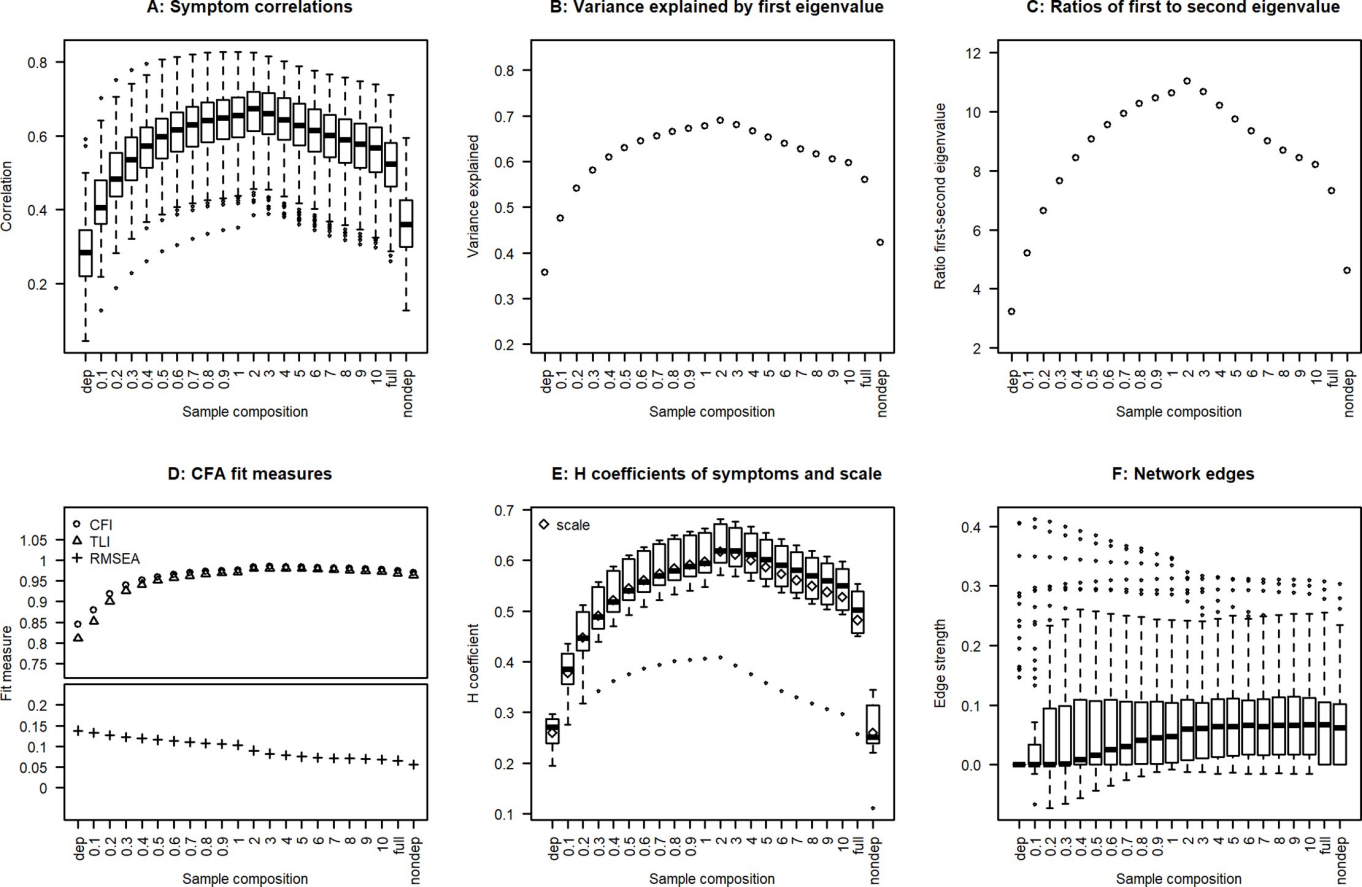
Correlations, dimensionality, scalability, and network edges of depression symptoms in function of the proportion of non-depressed participants in the study sample. A shows the correlations of the symptoms. B-C show the results of a principal component analysis. D shows the fit indices of a confirmatory one-factor analysis. E shows the scalability coefficient H for both the individual symptoms and the scale made up of all symptoms. F shows the edges of the symptom network. Abbreviations: dep: Depressed subjects only; full: General population sample, nondep: Non-depressed subjects only; CFA: Confirmatory Factor Analysis; CFI: Comparative Fit Index; TLI: Tucker-Lewis Index; RMSEA: Root Mean Square Error of Approximation.

The lowest symptom correlations were identified in the depressed-only sample (Fig 1A, average correlation = 0.29). All other samples, including the full population sample, exhibited higher symptom correlations. Adding as few as 10% of non-depressed subjects was accompanied by a clear-cut increase in the correlations’ strength (average correlation = 0.42). The impact was not strictly monotonic. Instead, correlations peaked in the sample containing twice as many non-depressed as depressed subjects (average correlation = 0.65) and then decreased again. The decrease was smaller than the increase, however. In particular, correlations were still much higher in the general population as compared to the depressed sample (average correlation = 0.51 versus 0.29).

### Dimensionality analysis

Parallel analysis revealed two dimensions in the depressed-only sample and one dimension in all other samples (results not shown). The dimensionality indicators exhibited the same pattern across proportions of non-depressed participants, as was found for the symptom correlations (Fig 1B and 1C):

- the lowest indicator values were found in the depressed-only sample (variance explained by first eigenvalue = 0.36, ratio first/second eigenvalue = 3.2)
- adding 10% of non-depressed participants produced a marked increase in both indicators (variance explained by first eigenvalue = 0.48, ratio first/second eigenvalue = 5.2) and the indicators were higher in the general population sample as in the depressed sample (variance explained by first eigenvalue: 0.56 versus 0.36, ratio first/second eigenvalue: 7.3 versus 3.2)
- the impact of the proportion of non-depressed subjects was again non-monotonic, with the highest indicator being found in the sample containing twice as many non-depressed as depressed subjects (variance explained by first eigenvalue = 0.69, ratio first/second eigenvalue = 11.0).

Considering the CFA fit measures, CFI and TLI also sharply increased as soon as non-depressed participants were added (Fig 1D). The CFI increased from 0.85 in the depressed sample to 0.97 in the general population sample. The corresponding increase in the TLI was from 0.81 to 0.97. In line with this, RMSEA decreased with an increasing proportion of non-depressed participants from 0.14 in the depressed to 0.07 in the general population sample, although this decline was less pronounced as the increases in CFI and TLI. Since 1-factor CFAs were estimated, the changes in fit measures imply that the factor structure became more unidimensional. However, rather than exhibiting the non-monotonic relationship described above, the fit measures ultimately plateaued.

### Mokken scale analysis

The H coefficients of scalability for both the symptoms and the entire scale exhibited the same pattern as the correlation coefficients (Fig 1E), specifically:

- the lowest H coefficients were found in the depressed-only sample (H of scale = 0.26);
- adding 10% of non-depressed participants produced a marked increase in the H coefficients (H of scale = 0.38), and the H coefficients were larger in the general population sample as in the depressed sample (H of scale = 0.48 versus 0.26)
- again a non-monotonic relationship existed between the proportion of non-depressed participants and the H coefficients.

Thus, the scalability of the symptoms was better with an increasing proportion of non-depressed participants and it was better in the general population as in the depressed sample.

### Network analysis

Considering the median of network edges, the edges tended to increase in magnitude with an increasing proportion of non-depressed subjects (Fig 1F). The increase was less pronounced as compared to correlations, however. This might be due to the fact that network edges were fairly small in magnitude, with a substantial portion of the edges being smaller than 0.10 in all samples. An increase was also present on the lower end of the edge distribution: samples containing a smaller proportion of non-depressed subjects produced larger negative edges. On the other hand, the largest positive edges became smaller in samples with increasing proportions of non-depressed subjects.

### Summary

To summarize, large and substantial changes were induced by changing the proportion of non-depressed participants in all psychometric analyses. To illustrate, when considering first the depressed sample and then the general population sample:

- the average symptom correlation increased from 0.29 to 0.51
- the variance explained by the first eigenvalue increased from 0.36 to 0.56
- the ratio of the first to the second eigenvalue increased from 3.2 to 7.3
- CFI and TLI increased from 0.85 to 0.97 and from 0.81 to 0.97, respectively, while the RMSEA decreased from 0.14 to 0.07
- the H coefficient of the entire scale increased from 0.26 to 0.48
- the median network edge strength increased from 0.00 to 0.07.

## Discussion

This investigation started with the curious finding that the strength and dimensionality of depression symptoms’ inter-relationships seem to change as a function of the dispersion of depression in the study sample [1–3, 6]. Consistent with the study hypotheses, it was found that the proportion of non-depressed participants in the study sample explained at least some of these findings: with higher proportions, correlations were stronger, network edges were stronger, unidimensionality was higher, and scalability was better. In fact, adding as few as 36 non-depressed to 356 depressed subjects (10%) had a major impact on the psychometric analyses. The findings thereby imply that results of psychometric analyses vary substantially as a function of the proportion of non-depressed participants in a study’s sample. This provides an explanation for the lack of reproducibility of previous psychometric studies [1].

In addition, these findings provide a potential explanation why psychometric results differed to such an extent when the baseline versus end-of-trial data of one and the same patient sample were compared in previous studies [2, 3]. Obviously, over the course of any trial, an increasing number of patients tend to remit. As a result, the sample composition changes such that the proportion of non-depressed subjects is zero at the beginning and becomes increasingly larger. Based on the current findings, it can be assumed that this change in sample composition produces a change from lower towards higher symptom correlations and an according increase in unidimensionality and scalability. Such a change was indeed documented by the afore-mentioned studies. This interpretation is further backed up by Sayer et al. who found that the correlation between two depression scales increased substantially during clinical trials. When they restricted the analysis to patients who remained substantially symptomatic during the trial on either scale, however, no such increase occurred [4].

### Why does the proportion of non-affected participants have such a strong influence?

A likely explanation is collider bias. Collider bias occurs if two variables A and B are causes of a variable C and the relationship between A and B is estimated while conditioning on C [31, 32]. Conditioning on C might be induced by statistically controlling for C, by stratifying on different levels of C or by using a sample selected based on C (such as using only a sample of subjects with high values of C) [31, 32].

Analyzing depression symptoms among depressed subjects corresponds to such a situation, because the depression sum score that underlies the depressed versus non-depressed classification is generated by the individual symptoms. Selecting depressed subjects therefore implies conditioning the subsequent analysis on the sum score. Adding more and more non-depressed individuals implies that the unselected sample is approximated and, hence, less conditioning on the sum score occurs. The proportion of non-depressed subjects likely acted as an index of how much conditioning on the sum score took place and hence, of the extent of collider bias.

If causes A and B are both positively related to C (which is the case with depression symptoms and the corresponding sum score), conditioning on C often attenuates the relationship between A and B and eventually even bias it towards a negative relationship [31, 32]. This is in line with the finding that adding non-depressed subjects to the depressed sample increased the magnitude of the symptom correlations. In the depressed sample, conditioning on the sum score attenuated the correlations, while with less conditioning in samples that contained increasing amounts of non-depressed subjects, the bias was reduced.

The reported results imply a more complicated picture, however. Although correlations, unidimensionality and scalability were substantially higher in the general population sample versus the depressed sample, they were highest in the sample with approximately twice as many non-depressed as depressed subjects. This is not in line with the classic notion of collider bias outlined above, because this notion implies that correlations should be highest in the general population sample and attenuated in selective samples. Additional research is needed to replicate and further elucidate this finding.

### Implications

The reported results raise a big question: which sample composition is the appropriate one on which to perform proper psychometric evaluations of the depression syndrome? More specifically, is there an appropriate number–or a minimal number—of non-depressed subjects that should be included in a sample to obtain an appropriate psychometric analysis of a depression measure?

To start with, it seems unlikely that the problem can be circumvented by selecting depressed subjects using a depression scale or clinical interview other than the one that provides the symptom data. The reason is that scale 1 is a proxy of scale 2 and vice versa. They have to be, since otherwise it would not be justified conceptually to say that depressed subjects were selected and depression symptoms were analyzed. However, in this situation, conditioning on depression scale 1 is highly redundant with conditioning on depression scale 2 and hence the problem is reproduced. Indeed, it was noted that conditioning on a proxy of a collider has the same consequence as conditioning on the collider itself [31]. In agreement with this, previous studies indicate that correlations between different depression scales increased substantially during clinical trials [4, 5], mirroring the increase of symptom correlations on the scale-level [2, 3]. However, additional studies are needed to establish the conjecture empirically.

Considering collider bias, the suggested remedy is to avoid conditioning on the collider [31]. This implies that the appropriate sample for psychometric analyses are samples without selection, that is, general population samples. Interestingly, research with such samples has revealed rather consistently that depression is unidimensional, whereas no consistent results were found in clinical samples [1]. The current results replicate the former finding and show that the scalability of symptoms is much higher in the general population as well. As a consequence, the psychometric problems might actually be smaller than initially thought because psychometric analyses in general population samples produce reasonable and replicable results. This is in agreement with the suggestion that—despite shortcomings—criteria of major depression do a reasonable job in their primary task: identifying depressed individuals [33].

In contrast, psychometric analyses in clinical samples are prone to produce unreliable results and may be avoided altogether. Focusing instead on specific, idiosyncratic symptom manifestations is more appropriate in these samples. Such a prioritization of symptoms over global depression scores is encouraged by the observation that common symptom lists do not cover the full range of clinically observed symptoms [33, 34], that depression symptoms have different risk factors [35–38]; by the large variability of symptom profiles found among patients [39, 40]; by the finding that symptoms differentially predict outcomes [41, 42]; and by the finding that symptoms are differentially impacted by antidepressant treatment [43, 44].

### Limitations

While the importance of sample composition was demonstrated, several issues should be addressed in future research. First, a major limitation is that the study was restricted to young men and to one cross-sectional assessment. As such, the results should merely be considered a starting point. Whether they generalize to more diverse samples and to patient samples monitored over the course of a clinical trial must be left to future studies. Note, however, that there are good reasons to assume that they will generalize, since they are consistent with previous results obtained across a variety of samples. Second, whereas collider bias certainly plays an important role, it does not readily explain the non-monotonic impact of the proportion of non-depressed. Future efforts should elucidate the effect of different proportions on collider bias more thoroughly. Third, it was proposed in the discussion section that the use of two different depression instruments—one for selecting depressed versus non-depressed subjects and one for obtaining symptom data—would reproduce collider bias. Future research should test this assumption empirically, since this could not be done with the data used in the current study. Finally, the interplay between general population and clinical samples in the psychometric study of major depression should be further elaborated.

### Conclusions

The reported results imply that psychometric analyses vary systematically as a function of the proportion of non-depressed subjects in the study sample. Therefore, psychometric analyses are not replicable across samples with different proportions of non-depressed subjects. This likely holds equally when analyzing clinical samples at the beginning versus the end of a clinical trial. Psychometric analyses should be carried out in general population samples.
